# Retention and Acceptability of a Linkage-to-Care Intervention Among Patients with Chronic Conditions in Rural South Africa

**DOI:** 10.3390/ijerph23050552

**Published:** 2026-04-24

**Authors:** Motlatso Elias Letshokgohla, Reneilwe Given Mashaba, Cairo Bruce Ntimana, Eric Maimela

**Affiliations:** 1 Department of Public Health, University of Limpopo, Sovenga St., Polokwane 0727, South Africa; letshokgohla@gmail.com; 2 DIMAMO Population Health Research Centre, University of Limpopo, Sovenga St., Polokwane 0727, South Africa; given.mashaba@ul.ac.za; 3 Department of Public Health, Walter Sisulu University, Butterworth 4960, South Africa; emaimela@wsu.ac.za

**Keywords:** linkage to care, acceptability, retention in care

## Abstract

**Highlights:**

**Public health relevance—How does this work relate to a public health issue?**

- Addresses poor retention in care among patients with chronic conditions (HIV, hypertension, and diabetes) in rural South Africa.
- Examines linkage-to-care interventions in low-resource settings where continuity of care remains a major public health challenge.

**Public health significance—Why is this work of significance to public health?**

- Identifies psychosocial factors (emotional engagement and self-efficacy) as key determinants of retention beyond traditional structural barriers.
- Demonstrates important differences in acceptability and retention across diagnostic groups, particularly among patients with multimorbidity.

**Public health implications—What are the key implications or messages for practitioners, policy makers and/or researchers in public health?**

- Linkage-to-care interventions should integrate psychosocial support and be tailored to patients’ daily realities, especially for those with multimorbidity.
- Policies and programs should move beyond one-size-fits-all approaches and prioritize patient-centered, context-specific care models to improve retention.

**Abstract:**

The prevalence of chronic conditions such as hypertension, diabetes, and Human Immunodeficiency Virus (HIV) is rising globally, yet access to continuous care remains limited, particularly in rural low- and middle-income countries. This study evaluated the acceptability and psychosocial predictors of retention in a linkage-to-care (LTC) intervention for patients with chronic conditions in rural South Africa. We conducted a cross-sectional analytical study with a retrospective cohort component among 1673 patients diagnosed with hypertension, diabetes, and/or HIV in Limpopo Province, South Africa. Acceptability and psychosocial factors were assessed cross-sectionally using a theory-informed, interviewer-administered questionnaire between January and June 2024. Retention in care over the preceding six months (July–December 2023) was extracted from routine clinic records and classified as consistent (no gaps > 6 months between visits) or inconsistent (≥1 gap > 6 months. Logistic regression examined associations between psychosocial factors and retention outcomes, adjusting for age, gender, marital status, and diagnostic category. Overall, 25.1% of participants maintained consistent retention over six months, while 74.9% were retained inconsistently. Acceptability of the LTC intervention varied significantly by diagnosis (*p* < 0.001): 79.5% of participants with multimorbidity rated the intervention as acceptable compared to 54.9% with hypertension, 64.5% with diabetes, and 46.8% with HIV. However, only 12.8% of multimorbid participants agreed that intervention activities fit well with their daily lives. In adjusted analyses, participants who were not happy to participate had 85% lower odds of consistent retention (adjusted odds ratio [AOR] = 0.15, 95% CI: 0.09–0.22) and 7.2 times higher odds of inconsistent retention (AOR = 7.2, 95% CI: 4.8–10.9). Most participants supported de-identified data sharing, though privacy concerns were elevated among those with multimorbidity. Acceptability of LTC interventions differs by diagnosis, with multimorbid patients reporting poorer alignment with daily routines. Retention is strongly associated with emotional engagement and self-efficacy, suggesting that LTC interventions should integrate psychosocial support and be contextually adapted for multimorbid patients in rural settings.

## 1. Introduction

The prevalence of chronic conditions such as hypertension, diabetes, and HIV is on the rise globally [1,2,3]. These conditions pose a great challenge and burden on global health systems [1,2,3]. The burden of these conditions is most negatively noted in low-middle-income countries (LMICs) [4]. According to the World Bank, South Africa is classified as an LMIC [5]. The burden of these conditions has been reported in South Africa, with the highest prevalences reported in rural areas, which are characterized by limited access to resources and continuous health care [6,7]. The limited access to care may inhibit early diagnosis and sustained retention in care, the two factors that positively contribute to successful patient outcomes [6,7].

In health systems research, LTC refers to the process by which individuals identified with a health issue are linked to appropriate healthcare providers and assisted in initiating and maintaining continuous medical treatment [8,9]. This process goes outside referral or first enrollment and involves integrated evaluation, patient orientation, health information, and psychological counseling to ensure continuity of care [8].

LTC interventions are strategies that aim to bridge the gap between diagnosis and continuous clinical management [10]. LTC interventions involve patient navigation, health education, and psychosocial support designed to guide patients through the health system [11,12]. However, there remains a gap in the implementation of these interventions. For instance, rural areas report suboptimal success, characterized by high attrition rates and inconsistent engagement after the initial enrollment [13,14]. Traditionally, the focus of LTC interventions has been on overcoming logistical barriers such as transportation costs, clinic hours, and medication availability [14,15]. Although these factors are important, literature suggests that non-logistical, patient-centered factors are equally important for sustained engagement [14,15].

The non-logistical factors include acceptability and understanding of the intervention, LTC interventions [16,17]. Acceptability is the perception among the participants that treatment or intervention is appropriate and useful [16,17]. Thus, for the LTC intervention to be effective, it must be considered acceptable and take into consideration the socio-cultural contexts of the participants [18]. In addition, this includes its fit in daily lives, its ethical implications, and the quality of interpersonal interactions with program staff. Furthermore, understanding what drives retention is rarely assessed in LTC programs in rural Africa.

This gap is particularly significant for patients with multimorbidity, whose complex care needs and potential for treatment burden may make them more vulnerable to dropping out of standard, single-disease-focused interventions [10]. Similarly, patients with Human Immunodeficiency virus (HIV) may face unique stigma-related barriers that influence their perception of an intervention’s acceptability and ethical consequences [19]. Therefore, a one-size-fits-all approach is likely to fail.

Examining differences in intervention acceptability and retention by diagnostic category is essential because patients with different chronic conditions face distinct challenges. HIV-positive patients may contend with stigma and confidentiality concerns that shape their perceptions of intervention appropriateness [19]. Patients with multimorbidity, the coexistence of two or more chronic conditions, face fragmented care systems and complex treatment regimens that may reduce intervention fit and increase treatment burden [20,21]. Understanding these condition-specific differences can inform targeted adaptations to LTC interventions, moving beyond one-size-fits-all approaches. Therefore, this study stratified analyses by hypertension, diabetes, HIV, and multimorbidity to identify differential patterns in acceptability, retention, and data-sharing preferences.

Therefore, this study aimed to evaluate the acceptability, key influencing factors, and perceived risks of an LTC intervention for patients with chronic conditions and to identify psychosocial predictors of patient retention over six months

## 2. Materials and Methods

### 2.1. Study Design

This employed a cross-sectional analytical design with a retrospective cohort component. The cross-sectional component assessed acceptability and psychosocial factors at a single time point (January–June 2024) using an interviewer-administered questionnaire. Retention in care was evaluated retrospectively over the preceding six-month period (July–December 2023) using routinely collected program records, including clinic attendance registers and electronic databases. Consequently, psychosocial factors were measured after the retention period; therefore, all associations should be interpreted as correlational rather than causal. The study was conducted at the DIMAMO Population Health Research Centre (PHRC). The DIMAMO PHRC is a Health and Demographic Surveillance System (HDSS) covering approximately 100,000 people, including all villages under the leadership of Tribal Authorities of Dikgale, J Mamabolo, A Mamabolo, and Mothiba in Capricorn District, Limpopo Province, South Africa. The LTC intervention comprised patient navigation, health education, follow-up support, and psychosocial engagement delivered alongside routine clinic-based care. Participants continued to receive condition-specific clinical services at their usual healthcare facilities, while the LTC intervention aimed to reduce disengagement by improving understanding of care pathways, supporting adherence-related behaviors, and enhancing retention in care. LTC was assessed through measures of retention over a six-month period and participants’ acceptability and psychosocial responses to the intervention, rather than through clinical integration.

### 2.2. Selection Criteria

All patients diagnosed with hypertension, diabetes, or HIV who are on Antiretroviral Therapy (ART) and attending clinics within the DIMAMO PHRC study area were included in this study. Patients with these diagnoses attending clinics in the study area were eligible. However, individuals unwilling to participate or those with mental health issues were excluded. This included participants experiencing distress or exhibiting behaviors that hindered their ability to function. This exclusion was implemented to ensure ethical participation and reliable assessment of intervention acceptability and retention, as severe psychological distress may independently affect engagement with care, irrespective of the LTC intervention. In this study, multimorbidity was defined as the coexistence of two or more chronic conditions (namely, hypertension, diabetes, and/or HIV). Participants were classified as having multimorbidity if they had at least two of these conditions documented in their clinic records and/or confirmed through self-report at the time of data collection

### 2.3. Participants and Sampling

The study population consisted of patients aged 18 years and older diagnosed with hypertension, diabetes, or HIV. Simple random sampling was utilized to select participants. A total of 1696 participants were sampled from the eight clinics. Of these, 1673 completed all data collection procedures (98.6% response rate). The data collection tool to assess the acceptability of LTC intervention by patients (recipients) was adapted from Sekho et al. [22].

### 2.4. Data Collection

Data were collected between 20 January and 30 June 2024 across eight clinics within the DIMAMO PHRC study area. A total of 1673 participants completed the data collection procedures, representing all individuals who provided informed consent. Participant recruitment and approach. Eligible patients attending routine clinic visits were identified from clinic registers based on diagnosis (hypertension, diabetes, and/or HIV). Research nurses, who were not involved in participants’ routine clinical care, approached potential participants in clinic waiting areas prior to or immediately after their scheduled consultations. The study purpose, procedures, risks, and benefits were explained in plain language (English, translated into Sepedi and Xitsonga by bilingual research staff). Willing individuals provided written informed consent before any data collection activities commenced.

Interviewer training and questionnaire administration. Four research assistants (fieldworkers) with prior experience in health surveys underwent a two-week training workshop. Training covered: (1) familiarization with the Theoretical Framework of Acceptability (TFA) constructs, (2) standardized administration of Likert-type questions, (3) cultural sensitivity and neutral probing techniques, (4) data confidentiality and privacy protocols, and (5) role-play scenarios with pilot testing (*n* = 20 participants, data not included in final analysis). Inter-rater reliability was assessed during piloting, with agreement exceeding 90% on all items.

All interviews were conducted in person using a structured, interviewer-administered questionnaire adapted from the Theoretical Framework of Acceptability (TFA) developed by Sekhon et al. [22]. Interviews took place in private rooms within the participating clinics to ensure confidentiality and minimize interruptions. No healthcare providers or clinic staff were present during interviews to reduce social desirability bias. Audio recording was not used; instead, research assistants recorded participant responses directly onto paper questionnaires. The questionnaire assessed acceptability domains relevant to LTC interventions, including affective attitude, intervention coherence, self-efficacy, ethicality and fairness, and perceived fit and social support. Responses were recorded using Likert-type scales ranging from *strongly disagree* to *strongly agree*, and from *very unacceptable* to *very acceptable* for overall acceptability. The questionnaire underwent contextual adaptation through expert review by public health researchers and LTC practitioners familiar with the rural Limpopo setting to ensure clarity, cultural relevance, and appropriateness of terminology. Minor wording adjustments were made to reflect local healthcare delivery processes. Content validity was established through this expert review process. Internal consistency reliability was assessed using Cronbach’s alpha for each acceptability domain. Interviewers were instructed not to paraphrase or interpret responses but to read items exactly as written and record the participant’s selected option. At the end of each day, completed questionnaires were reviewed for completeness by a senior researcher, and any missing or ambiguous entries were flagged for follow-up with the participant where possible (within 48 h).

The average interview duration was 25 min (range: 18–40 min), varying by participant literacy level and need for translation between English and local languages (Sepedi or Xitsonga).

Additional items assessed health data privacy and sharing, including comfort with electronic health data access, perceived risks of re-identification, and willingness to share de-identified data, informed by prior studies on confidentiality in chronic and HIV care [23].

Retention data. Retention outcomes for the six-month period July–December 2023 were extracted retrospectively from routine clinic attendance registers and electronic program databases. These data were collected by clinic staff as part of standard care and were not influenced by the research team. Research assistants abstracted visit dates, diagnostic codes, and attendance patterns using a standardized data extraction form. Double-data entry was performed on a 10% random sample to verify accuracy (error rate < 1%). Retention outcomes were classified as retention without gaps > 6 months or inconsistent retention with gaps > 6 months.

### 2.5. Variable Definitions

The dependent variable, retention-to-care (RTC), was classified into two mutually exclusive categories based on clinic attendance over a six-month follow-up period (July–December 2023): consistent retention, defined as having at least one documented clinic visit with no interval exceeding six months between consecutive visits, and inconsistent retention, defined as having at least one interruption in care where the interval between visits exceeded six months. The independent variables included acceptability domains measured using a theory-informed questionnaire adapted from Sekhon et al., capturing affective attitude, intervention coherence, self-efficacy, ethicality, and perceived fit; diagnostic categories comprising hypertension only, diabetes only, HIV only (on ART), and multimorbidity (defined as the coexistence of two or more of these conditions based on clinic records and/or self-report); and psychosocial factors such as emotional engagement (e.g., happiness to participate and enjoyment of discussions), self-efficacy (confidence in maintaining new habits), and perceived support. Covariates included age (categorized as 18–34, 35–44, 45–54, 55–64, and ≥65 years), gender (male/female), and marital status (never married, married, divorced, or widowed).

### 2.6. Outcome Definition

RTC outcomes were determined retrospectively using routine clinic attendance records and LTC program databases. Two mutually exclusive RTC categories were defined. “RTC without gaps longer than six months” was defined as continuous engagement in care during the six-month follow-up period, with at least one documented clinic visit and no interval exceeding six months between any two consecutive visits. “Inconsistent RTC with gaps longer than six months” was defined as the presence of at least one interruption in care in which the interval between consecutive clinic visits exceeded six months during the same follow-up period. Visit dates were extracted from clinic registers and electronic program records, which served as the primary data sources for retention classification

### 2.7. Statistical Analysis

Data were analyzed using the Statistical Package for the Social Sciences (SPSS) version 28 (I.B.M., Armonk, New York, NY, USA). Categorical variables were presented in percentages and frequencies, and continuous variables were presented in terms of means and percentages. A Chi-Square test was used to compare proportions among groups. Multivariable logistic regression models were fitted to examine associations between each psychosocial factor and retention outcomes, adjusting for potential confounders including age, gender, marital status, and diagnostic category. Adjusted odds ratios (AORs) with 95% confidence intervals were calculated. For each factor, the response “Yes” served as the reference category, and the AORs represent the likelihood of the outcome among participants responding “No” compared to those responding “Yes.” A *p*-value of less than 0.05 was considered statistically significant

## 3. Results

The present study analysed 1673 patients diagnosed with either HIV (on ART), hypertension, or diabetes. The mean age of the participants was 50.47 ± 17.05 years, with most of the participants being females. Most of the female participants were never married as compared to males. The distribution of patients diagnosed with hypertension, diabetes, and/or HIV on ART varied by gender. A higher proportion of females (30.9%) were diagnosed with hypertension compared to males (22.8%). In contrast, diabetes was more prevalent among males (35.8%) than among females (30.5%). Similarly, more females (23.8%) were diagnosed with HIV than males (21.6%). Additionally, a greater proportion of males (19.9%) were diagnosed with both hypertension and diabetes, compared to females (14.8%), as shown in Table 1.

Among the 1673 individuals aged ≥18 years or older registered in the LTC intervention in the eight clinics, 1253 (74.9%) were retained in care but not consistent during a period of six months, while 420 (25.1%) were retained in care and consistent within a period of six months. The acceptability of the LTC intervention varied significantly by patient diagnosis (*p* < 0.001 for all measures). Most participants indicated that the activities fitted well with how they wanted to live their lives, with 24.3% of those with hypertension, 28.3% with diabetes, 26.9% with HIV, and 12.8% with multimorbidity agreeing. Similarly, many valued support from people other than healthcare providers, including 29.2% with hypertension, 31.9% with diabetes, 22.3% with HIV, and 16.0% with multimorbidity (*p* < 0.001). Regarding overall acceptability, 54.9% of participants with hypertension, 64.5% with diabetes, 46.8% with HIV, and 79.5% with multimorbidity rated the intervention as acceptable, while 43.6%, 33.5%, 49.9%, and 20.5%, respectively, rated it as very acceptable (*p* < 0.001).

When asked about the moral or ethical consequences of participating in the intervention, the majority agreed or strongly agreed, with 63.4% and 25.0% of those with hypertension, 64.7% and 20.1% with diabetes, 48.3% and 38.8% with HIV, and 73.5% and 9.7% with multimorbidity agreeing (*p* < 0.001). In terms of fairness, participants generally perceived the intervention as fair, with 65.9% of those with hypertension, 68.6% with diabetes, 55.3% with HIV, and 66.0% with multimorbidity rating it as fair, while 21.3%, 16.4%, 33.2%, and 9.7%, respectively, rated it as very fair (*p* < 0.001) (Table 2).

Table 3 presents participants’ attitudes toward data privacy and sharing across individuals diagnosed with hypertension, diabetes, HIV, and those with multimorbidity. Overall, most respondents expressed comfort with researchers accessing their electronic health data, though the degree of comfort varied by condition. For example, nearly half of respondents with multimorbidity (48.9%) “somewhat agreed” with non-care researchers accessing their data, compared to 42.8% of hypertensive and 43.8% of diabetic participants. However, a smaller proportion “completely agreed,” particularly among the multimorbidity group (16.0%), suggesting some hesitation. When asked about acquaintances (e.g., friends or coworkers) accessing their data for research, the levels of agreement declined, with only about 26–31% across all groups “completely agreeing.” Participants also expressed notable concerns about re-identification and disclosure risks, especially among those with multimorbidity, where more than half (51.5%) “agreed” that data disclosure to employers or people they know poses risks (*p* < 0.001). Despite these concerns, strong support emerged for confidential data sharing when identifiers were removed: 55.2% of multimorbid participants and roughly half of those with hypertension (46.9%) and diabetes (50.4%) “somewhat agreed,” while 36–42% “completely agreed” across all conditions.

Table 4 presents adjusted associations between psychosocial factors and retention outcomes from multivariable logistic regression models controlling for age, gender, marital status, and diagnostic category. Participants who reported not being happy to participate in the LTC intervention had 85% lower odds of consistent retention compared to those who were happy (AOR = 0.15, 95% CI: 0.09–0.22, *p* < 0.001). Conversely, they had 7.2 times higher odds of inconsistent retention (AOR = 7.2, 95% CI: 4.8–10.9, *p* < 0.001).

Similarly, those who did not enjoy discussions with the facilitator had 58% lower odds of consistent retention (AOR = 0.42, 95% CI: 0.30–0.58, *p* < 0.001) and 2.1 times higher odds of inconsistent retention (AOR = 2.1, 95% CI: 1.7–2.6, *p* < 0.001). Participants who did not appreciate the suggested activities had 62% lower odds of consistent retention (AOR = 0.38, 95% CI: 0.28–0.48, *p* < 0.001) and 2.6 times higher odds of inconsistent retention (AOR = 2.6, 95% CI: 2.1–3.3, *p* < 0.001). Participants who did not enjoy working with care companions had 82% lower odds of consistent retention (AOR = 0.18, 95% CI: 0.12–0.24, *p* < 0.001) and 5.4 times higher odds of inconsistent retention (AOR = 5.4, 95% CI: 4.2–6.9, *p* < 0.001).

Difficulty understanding how the intervention could help was associated with 42% lower odds of consistent retention (AOR = 0.58, 95% CI: 0.42–0.75, *p* < 0.001) and 1.7 times higher odds of inconsistent retention (AOR = 1.7, 95% CI: 1.4–2.1, *p* < 0.001). Participants who reported not spending much time with healthcare workers due to the intervention had 52% lower odds of consistent retention (AOR = 0.48, 95% CI: 0.36–0.62, *p* < 0.001) and 2.1 times higher odds of inconsistent retention (AOR = 2.1, 95% CI: 1.7–2.6, *p* < 0.001).

Lack of confidence in continuing new habits was strongly associated with poor retention. Participants who were not confident had 72% lower odds of consistent retention (AOR = 0.28, 95% CI: 0.19–0.38, *p* < 0.001) and 3.4 times higher odds of inconsistent retention (AOR = 3.4, 95% CI: 2.6–4.4, *p* < 0.001). Similarly, those who did not feel that intervention activities fit well with their lives had 69% lower odds of consistent retention (AOR = 0.31, 95% CI: 0.22–0.42, *p* < 0.001) and 3.4 times higher odds of inconsistent retention (AOR = 3.4, 95% CI: 2.7–4.3, *p* < 0.001).

Not valuing support from others besides healthcare providers was associated with 68% lower odds of consistent retention (AOR = 0.32, 95% CI: 0.21–0.45, *p* < 0.001) and 3.7 times higher odds of inconsistent retention (AOR = 3.7, 95% CI: 2.3–6.0, *p* < 0.001).

Two variables were not significantly associated with retention outcomes: perceiving sufficient information about the intervention (consistent retention AOR = 0.85, 95% CI: 0.68–1.06; inconsistent retention AOR = 1.2, 95% CI: 0.9–1.4) and making schedule changes to attend sessions (consistent retention AOR = 0.92, 95% CI: 0.73–1.15; inconsistent retention AOR = 1.1, 95% CI: 0.9–1.3).

## 4. Discussion

The present study aimed to evaluate the acceptability, key influencing factors, and perceived risks of an LTC intervention for patients with chronic conditions. This intervention was intentionally designed by adapting established HIV linkage strategies used in South Africa, such as telephone follow-up, home visits, and community-based support, to a multi-condition context, thereby offering a clearly defined and replicable protocol within an otherwise heterogeneous integrated HIV–NCD evidence base. The findings of the present study demonstrated that although overall acceptability of the intervention was high, perceptions and behavioral responses differed by diagnosis, emotional engagement, and perceived relevance. Of LTC interventions. The findings further revealed that participants’ retention in LTC interventions was shaped by structural or logistical factors as well as by affective, ethical, and relational dimensions of care.

The present study found that the acceptability of the LTC intervention was high across all groups. However, the degree of this acceptance differed by diagnosis. For instance, participants with HIV expressed the highest proportion of acceptance. Meanwhile, those with multimorbidity expressed the lowest levels of enthusiasm. They further reported that the interventions did not fit well with their daily lives. Although participants with multimorbidity reported high overall acceptability scores, their agreement that the intervention activities fitted well with their daily lives and support systems was notably lower. This suggests that their acceptance may have been more nominal than experiential, reflecting limited emotional engagement and poor integration with complex care routines. This finding mirrors those of studies conducted in Africa and globally [20,24]. For instance, a study conducted in Tanzania found high levels of satisfaction with LTC interventions amongst individuals living with HIV [25]. This was due to the incorporation of structured psychosocial and stigma reduction components into the LTC interventions [25]. On the other hand, Khatib et al. [26] reported that patients’ multimorbidity found LTC interventions lack integration of services for multiple chronic conditions. Thus, patients with single chronic conditions experience alignment between intervention activities and needs. However, multimorbid patients face fragmented care systems and complex treatment regimens [27]. Several studies observed that LTC interventions designed for single diseases are not effective in patients with multimorbidity [20,21].

The present study found that retention in care intervention was influenced by emotional engagement and self-efficacy than by logistical convenience. In adjusted analyses controlling for demographic factors and diagnostic category, patients who reported not enjoying the intervention or felt emotionally disconnected from facilitators were between 5.4 and 7.2 times more likely to be inconsistently retained. Similarly, those who lacked confidence in sustaining new habits were 3.4 times more likely to drop out. According to the literature, behavior change interventions are sustained not only by information provision but also by affective motivation and belief in self-efficacy [28,29,30]. In chronic disease management, self-efficacy-enhancing interventions improve medication adherence and long-term retention [28,29,30]. The current study extends this evidence to a broader multimorbidity context by highlighting that affective engagement functions as a key determinant of sustained participation. Of note, these findings contrast with older linkage models where retention was conceptualized primarily as a structural or access issue (clinic distance, cost, or appointment scheduling) [31,32]. In the present study, emotional and relational experiences proved more predictive than logistical barriers. This aligns with Lee et al., who reported that communication quality, empathy, and relational trust are central to adherence [33]. Most participants were willing to share de-identified health data for research. However, some participants raised privacy concerns, with those in the multimorbidity group showing more concern. Patients with multimorbidity are reported to have more data sharing concerns over potential confidentiality breaches due to overlapping care systems [34]. In contrast, HIV patients have been reported to have increased comfort with data sharing, reflecting normalization of confidentiality frameworks within HIV programs [35].

The present study found that participants acknowledged the moral and ethical implications of participating in the LTC intervention. However, those in the HIV positive category reported the strongest agreement. The findings of the present study highlight an ongoing moralization of HIV care despite governmental efforts to de-stigmatize HIV [36]. Studies conducted in Sub-Saharan Africa found that HIV stigma continues to influence HIV care programs [37,38,39]. This influence further shapes how patients perceive confidentiality, self-worth, and morality [38,39,40]. Patients living with HIV are reported to internalize societal judgments often and perceive engagement in HIV care as both an ethical obligation and a risk of moral exposure [40]. For non-communicable disease (NCD) patients, ethical framing is likely due to perceived personal responsibility for health. This trend was also observed by Audulv et al. [41], in a study that reported that chronic disease interventions often trigger self-blame narratives linked to lifestyle management.

### Limitations and Strengths of the Study

Several limitations should be considered when interpreting these findings. First, the cross-sectional design and retrospective assessment of retention preclude causal inference; psychosocial factors were measured after the retention period, making the temporal direction of associations unclear. Longitudinal studies with baseline psychosocial measurement are needed to establish causality. Second, individuals with mental health conditions were excluded, which may have introduced selection bias and limited generalizability to this high-risk population. Third, self-reported acceptability data may be subject to social desirability bias. Fourth, despite adjusting for demographic factors and diagnostic category, residual confounding from unmeasured variables such as socioeconomic status, distance to clinic, and health literacy cannot be excluded. Fifth, the single-site design in rural Limpopo may limit generalizability to other settings. Sixth, retention classification based on clinic attendance records may not fully capture the complexity of patient engagement.

Despite these limitations, the study has important strengths, including a large sample size (*n* = 1673), multivariable adjustment for confounders, stratification by diagnostic category to identify condition-specific patterns, and novel contributions regarding patient perceptions of data privacy in the context of linkage-to-care interventions for chronic conditions in rural Africa.

## 5. Conclusions

The present study showed that while the LTC intervention was generally acceptable among patients with chronic conditions, perceptions of its relevance and impact varied by diagnosis. Acceptability ratings were highest among HIV-positive participants, whereas individuals with multimorbidity exhibited lower emotional enthusiasm and less alignment between the intervention and their daily routines. Consistent retention was more strongly associated with emotional engagement and self-efficacy than with logistical convenience, highlighting the importance of motivation, trust, and relational continuity as factors correlated with sustained participation. Although most participants were willing to share de-identified health data, concerns about privacy and confidentiality were greater among those with multimorbidity. Ethical and moral perceptions also differed by diagnosis, with HIV-positive participants reporting stronger agreement regarding the moral implications of engagement, reflecting ongoing stigma and moralization of care.

Overall, the findings underscore the need for emotionally engaging, contextually adaptive, and integrated LTC interventions to improve retention and acceptability among patients with chronic conditions in rural South Africa. Based on the findings of the present study, future research using longitudinal and mixed-methods designs is needed to examine causal pathways between psychosocial factors, acceptability, and long-term retention in LTC interventions. There is also a need to develop and evaluate integrated LTC models that explicitly address multimorbidity, rather than relying on single-disease frameworks. In addition, qualitative studies exploring patient experiences, ethical concerns, and contextual influences in rural settings would provide deeper insight into mechanisms shaping engagement and sustained participation in care.

## Figures and Tables

**Table 1:** Characteristics of study participants by gender.

Characteristic	Total (*n* = 1673) *n* (%)	Females (*n* = 1260) *n* (%)	Males (*n* = 413) *n* (%)	*p*-Value
**Age in years**				<0.001
**18–34**	308 (18.4)	262 (20.8)	46 (11.1)	
**35–44**	395 (23.6)	321 (25.5)	74 (17.9)	
**45–54**	323 (19.3)	234 (18.6)	89 (21.6)	
**55–64**	211 (12.6)	155 (12.3)	56 (13.6)	
**≥65**	436 (26.1)	288 (22.9)	148 (35.8)	
**Marital status**				<0.001
**Never married**	1053 (62.9)	832 (66.0)	221 (53.5)	
**Married**	526 (31.4)	357 (28.3)	169 (40.9)	
**Divorced**	18 (1.1)	13 (1.0)	5 (1.2)	
**Widowed**	76 (4.5)	58 (4.6)	18 (4.4)	
**Diagnosis**				0.001
**Hypertension**	484 (28.9)	390 (30.9)	94 (22.8)	
**Diabetes**	532 (31.8)	384 (30.5)	148 (35.8)	
**HIV**	389 (23.3)	300 (23.8)	89 (21.6)	
**Multimorbidity**	268 (16.0)	186 (14.8)	82 (19.9)	

**Table 2:** Acceptability and ethical perceptions of the intervention.

Diagnosis	Hypertension *n* (%)	Diabetes *n* (%)	HIV *n* (%)	Multimorbidity *n* (%)	*p*-Value
**The acceptability of LTC intervention stratified by diagnosis**					
**Activities in this LTC intervention have fitted well with how I want to live my life**	321 (24.3)	284 (28.3)	270 (26.9)	140 (12.8)	<0.001
**The possibility of support from others besides healthcare providers is important for me**	469 (29.2)	513 (31.9)	358 (22.3)	268 (16.0)	<0.001
**Acceptability of the LTC intervention to participants stratified by diagnosis**					
**Very unacceptable**	3 (0.6)	0 (0.0)	3 (0.8)	0 (0.0)	<0.001
**Unacceptable**	2 (0.4)	3 (0.6)	3 (0.8)	0 (0.0)	
**No opinion**	2 (4)	8 (1.5)	7 (1.8)	0 (0.0)	
**Acceptable**	266 (54.9)	343 (64.5)	182 (46.8)	213 (79.5)	
**Very acceptable**	211 (43.6)	178 (33.5)	194 (49.9)	55 (20.5)	
**There are moral or ethical consequences to engaging in the LTC intervention**					
**Strongly disagree**	39 (8.1)	56 (10.5)	28 (7.2)	36 (13.4)	<0.001
**Disagree**	9 (1.9)	9 (1.7)	7 (1.8)	3 (1.1)	
**No opinion**	8 (1.7)	16 (3.0)	15 (3.9)	6 (2.2)	
**Agree**	307 (63.4)	344 (64.7)	188 (48.3)	197 (73.5)	
**Strongly agree**	121 (25.0)	107 (20.1)	151 (38.8)	26 (9.7)	
**Fairness of the LTC intervention**					
**Very unfair**	0 (0.0)	1 (0.2)	1 (0.3)	0 (0.0)	<0.001
**Unfair**	20 (4.1)	29 (5.5)	17 (4.4)	17 (6.3)	
**No opinion**	42 (8.7)	50 (9.4)	27 (6.9)	48 (17.9)	
**Fair**	319 (65.9)	365 (68.6)	215 (55.3)	177 (66.0)	
**Very fair**	103 (21.3)	87 (16.4)	129 (33.2)	26 (9.7)	

**Table 3:** Perceptions of data sharing and linkage risks.

	Hypertension *n* (%)	Diabetes *n* (%)	HIV *n* (%)	Multimorbidity	*p-Val* *ue for Trend*
**Comfortable with researchers not directly involved in my care accessing my electronic health data for research purposes**					
**Completely Disagree**	7 (1.5)	7 (1.3)	5 (1.3)	0 (0.0)	0.001
**Somewhat Disagree**	41 (8.5)	38 (7.1)	46 (11.8)	4 (1.5)	
**Moderately Agree**	145 (29.9)	153 (28.8)	106 (27.3)	90 (33.6)	
**Somewhat Agree**	207 (42.8)	233 (43.8)	154 (39.6)	131 (48.9)	
**Completely Agree**	84 (17.4)	101 (18.9)	78 (20.1)	43 (16.0)	
**Comfortable with someone I know (e.g., friend, neighbour, co-worker) who is a researcher accessing my electronic health data for research purposes**					
**Completely Disagree**	23 (4.8)	31 (5.8)	16 (4.1)	25 (9.3)	0.067
**Somewhat Disagree**	36 (7.4)	49 (9.2)	30 (7.7)	21 (7.8)	
**Moderately Agree**	129 (26.7)	121 (22.7)	118 (30.3)	80 (29.9)	
**Somewhat Agree**	158 (32.6)	169 (31.7)	124 (31.9)	72 (26.9)	
**Completely Agree**	138 (28.5)	162 (30.5)	101 (25.9)	70 (26.1)	
**Concerns about the potential risks associated with their health data being re-identified and disclosed to employers**					
**Strongly disagree**	24 (4.9)	27 (5.1)	24 (6.2)	2 (0.8)	0.001
**Disagree**	47 (9.1)	47 (8.8)	56 (14.4)	10 (3.7)	
**No opinion**	100 (20.7)	86 (16.2)	94 (24.2)	33 (12.3)	
**Agree**	201 (41.5)	233 (43.8)	140 (35.9)	138 (51.5)	
**Strongly Agree**	112 (23.1)	139 (26.1)	75 (19.3)	85 (31.7)	
**Concerns about the potential risks associated with my health data being re-identified and disclosed to people I know**					
**Strongly disagree**	26 (5.4)	22 (4.1)	22 (5.7)	2 (0.8)	0.001
**Disagree**	19 (3.9)	39 (7.3)	28 (7.2)	5 (1.9)	
**No opinion**	87 (17.9)	79 (14.9)	99 (25.5)	29 (10.8)	
**Agree**	223 (46.1)	248 (46.6)	144 (37.0)	137 (51.1)	
**Strongly Agree**	129 (26.7)	144 (27.1)	96 (24.7)	95 (35.5)	
**Concerns about the potential risks associated with my health data being disclosed to researchers or doctors not involved in my care**					
**Strongly disagree**	23 (4.8)	21 (3.9)	39 (10.0)	0 (0.0)	<0.001
**Disagree**	69 (14.3)	64 (12.0)	58 (14.9)	21 (7.8)	
**No opinion**	94 (19.4)	86 (16.2)	92 (23.7)	47 (17.5)	
**Agree**	194 (40.1)	214 (40.2)	121 (31.1)	138 (51.5)	
**Strongly Agree**	104 (21.5)	147 (27.6)	79 (20.3)	62 (23.1)	
**Concerns about the potential risks associated with my health data being re-identified and disclosed to people I don’t know**					
**Strongly disagree**	21 (4.3)	16 (3.0)	19 (4.9)	2 (0.8)	0.064
**Disagree**	116 (23.9)	134 (25.2)	88 (22.6)	66 (24.6)	
**No opinion**	164 (33.9)	163 (30.6)	105 (26.9)	99 (36.9)	
**Agree**	127 (26.2)	149 (28.0)	121 (31.1)	72 (26.9)	
**Strongly Agree**	56 (11.6)	70 (13.2)	56 (14.4)	29 (10.8)	
**Comfortable with health data being confidentially shared with researchers, as long as personal information is not provided**					
**Completely Disagree**	8 (1.7)	7 (1.3)	8 (2.1)	0 (0.0)	<0.001
**Somewhat Disagree**	23 (4.8)	15 (2.8)	24 (6.2)	0 (0.0)	
**Moderately Agree**	23 (10.1)	44 (8.3)	61 (15.7)	6 (2.2)	
**Somewhat Agree**	227 (46.9)	268 (50.4)	169 (43.4)	148 (55.2)	
**Completely Agree**	177 (36.6)	198 (37.7)	127 (32.7)	114 (42.5)	

**Table 4:** Retention predictors and psychosocial factors.

Psychosocial Factor	Category	Consistent Retention AOR (95% CI)	Inconsistent Retention AOR (95% CI)
**Affective Attitude Domain**			
**Happy to participate**	Yes (Ref)	1.00 (Ref)	1.00 (Ref)
	No	0.15 (0.09–0.22) ***	7.2 (4.8–10.9) ***
**Enjoy discussions with facilitator**	Yes (Ref)	1.00 (Ref)	1.00 (Ref)
	No	0.42 (0.30–0.58) ***	2.1 (1.7–2.6) ***
**Appreciate suggested activities**	Yes (Ref)	1.00 (Ref)	1.00 (Ref)
	No	0.38 (0.28–0.48) ***	2.6 (2.1–3.3) ***
**Enjoy working with care companions**	Yes (Ref)	1.00 (Ref)	1.00 (Ref)
	No	0.18 (0.12–0.24) ***	5.4 (4.2–6.9) ***
**Intervention Coherence & Self-Efficacy Domain**			
**Easy to understand how intervention can help**	Yes (Ref)	1.00 (Ref)	1.00 (Ref)
	No	0.58 (0.42–0.75) ***	1.7 (1.4–2.1) ***
**Received enough information about intervention**	Yes (Ref)	1.00 (Ref)	1.00 (Ref)
	No	0.85 (0.68–1.06)	1.2 (0.9–1.4)
**Changed schedule to attend sessions**	Yes (Ref)	1.00 (Ref)	1.00 (Ref)
	No	0.92 (0.73–1.15)	1.1 (0.9–1.3)
**Spent much time with healthcare workers**	Yes (Ref)	1.00 (Ref)	1.00 (Ref)
	No	0.48 (0.36–0.62) ***	2.1 (1.7–2.6) ***
**Confident in continuing new habits**	Yes (Ref)	1.00 (Ref)	1.00 (Ref)
	No	0.28 (0.19–0.38) ***	3.4 (2.6–4.4) ***
**Activities fit well with how I want to live**	Yes (Ref)	1.00 (Ref)	1.00 (Ref)
	No	0.31 (0.22–0.42) ***	3.4 (2.7–4.3) ***
**Support from others is important**	Yes (Ref)	1.00 (Ref)	1.00 (Ref)
	No	0.32 (0.21–0.45) ***	3.7 (2.3–6.0) ***

Values are adjusted odds ratios (AOR) with 95% confidence intervals. All models were adjusted for age, gender, marital status, and diagnostic category. The reference category for each factor is “Yes”. *** *p* < 0.001.

## Data Availability

The data presented in this study are available on request from the corresponding author. The data are not publicly available due to privacy or ethical restrictions.
